# Evaluating Oxidative Stress in Human Cardiovascular Disease: Methodological Aspects and Considerations

**DOI:** 10.2174/092986712800493057

**Published:** 2012-06

**Authors:** R Lee, M Margaritis, KM Channon, C Antoniades

**Affiliations:** Department of Cardiovascular Medicine, University of Oxford, Oxford, UK

**Keywords:** Analytical methods, atherosclerosis, biomarkers, lipid peroxidation, oxidative stress, nitric oxide, superoxide.

## Abstract

Oxidative stress is a key feature in atherogenesis, since reactive oxygen species (ROS) are involved in all stages of the disease, from endothelial dysfunction to atheromatic plaque formation and rupture. It is therefore important to identify reliable biomarkers allowing us to monitor vascular oxidative stress status. These may lead to improved understanding of disease pathogenesis and development of new therapeutic strategies.

Measurement of circulating biomarkers of oxidative stress is challenging, since circulation usually behaves as a separate compartment to the individual structures of the vascular wall. However, measurement of stable products released by the reaction of ROS and vascular/circulating molecular structures is a particularly popular approach. Serum lipid hydroperoxides, plasma malondialdehyde or urine F2-isoprostanes are widely used and have a prognostic value in cardiovascular disease.

Quantification of oxidative stress at a tissue level is much more accurate. Various chemiluminescence and high performance liquid chromatography assays have been developed over the last few years, and some of them are extremely accurate and specific. Electron spin resonance spectroscopy and micro-electrode assays able to detect ROS directly are also widely used.

In conclusion, measurement of circulating biomarkers of oxidative stress is valuable, and some of them appear to have predictive value in cardiovascular disease. However, these biomarkers do not necessarily reflect intravascular oxidative stress and therefore cannot be used as therapeutic targets or markers to monitor pharmacological treatments in clinical settings. Measurement of vascular oxidative stress status is still the only reliable way to evaluate the involvement of oxidative stress in atherogenesis.

## INTRODUCTION

Atherosclerosis is one of the major health problems worldwide, driving cardiovascular morbidity and mortality. The understanding of atherogenesis has dramatically improved over the last decade, allowing the identification of new therapeutic targets and the development of novel therapeutic strategies. Endothelial dysfunction is a first step in atherogenesis [1], and it is characterized by reduced nitric oxide (NO) bioavailability and increased generation of reactive oxygen species (ROS) in the vascular wall [2]. Oxidative stress is defined by the imbalance between the production of ROS and the endogenous antioxidant mechanisms to counteract the effects of ROS or to repair the resulting damages [3]. Under physiological conditions, several tightly controlled oxidative pathways contribute towards ROS productions, while several intra- and extra- cellular antioxidant enzymatic mechanisms account for ROS elimination [3]. Oxidative stress is therefore a critical feature in atherogenesis. ROS are responsible for direct damage to cellular structures within the vascular wall, while they also trigger a number of redox sensitive transcriptional pathways, shifting towards a pro-atherogenic transcriptomic profile. 

Low density lipoprotein (LDL) is the critical element in the prevailing theories of atherogenesis. Deposition of LDLs in the subendothelial space and the subsequent transcytosis by resident / circulating macrophages lead to formation of foam cells – the hallmark of atherosclerosis [4]. Oxidation of circulating LDLs particles occurs primarily at the vascular endothelium rather than in plasma, which is enriched with innate antioxidant defense mechanisms [5]. LDL becomes entrapped in the sub-endothelial space where it is subject to oxidative modification to form oxidized LDL (oxLDL). This is then readily internalized by macrophages through the “scavenger receptor” pathway originally described by Brown and Goldstein [6], leading to the accumulation of foam cells and subsequent atherogenesis [6].

Endothelium has been identified as a major source of ROS in the human blood vessels [7] and endothelial function is closely linked to homeostasis of ROS formation within the vascular wall [8]. Risk factors for atherosclerosis such as smoking, hypertension and diabetes mellitus, are associated with an increased production of ROS by the endothelium. As LDLs traverse the sub-endothelial space in lesion prone arterial segments, oxidative damage caused by these ROS occurs. This involves the process of lipid peroxidation and apolipoprotein B-100 modification, leading to the formation of oxLDL. oxLDLs are involved in many atherogenic steps in the vascular wall such as endothelial dysfunction, migration of macrophages and smooth muscle cells and release of inflammatory cytokines, and are avidly internalized by macrophages via scavenger-receptor pathways. In addition, oxLDL also induces further oxidative stress to the endothelial cells, smooth muscle cells and foams cells, thus perpetuating the cycle of atherogenesis. Further to its involvement in atherogenesis, circulating oxLDL has also been implicated in the pathogenesis of metabolic disorders such as obesity and diabetes mellitus [9]. The critical role of oxLDL in atherogenesis was highlighted by recent evidence based on apolipoprotein E (ApoE) deficient mice, which demonstrated that atherosclerotic progression could be completely inhibited by ectopic hepatic expression of lectin-like oxLDL receptor 1 (LOX-1). Decreased levels lipid peroxide and monocyte chemotactic protein-1 (MCP-1) were observed in the same mice, suggesting a positive effect on oxidative stress by selective clearance of oxLDL [10]. A schematic illustration which highlights the role of oxLDL throughout the stages of atherogenesis is shown in Fig. (**1**).

In accordance to its ubiquitous involvement in atherogenesis, there has been significant effort to evaluate the circulating oxLDL levels as a biomarker for the progression and severity of advanced atherosclerotic disease state such as coronary artery disease (CAD). In a prospective case control study involving 346 subjects, elevated plasma oxLDL level in apparently healthy men was associated with increased risk of an acute CAD event. After adjustment for the traditional risk factors of CAD, subjects in the highest tertile of plasma oxLDL has a significant hazard ratio (HR) of 4.25 for a future CAD event compared to subjects in the lower tertile. In this cohort, plasma oxLDL was a stronger predictor of future CAD events when compared to other traditional risk factors and conventional lipoprotein profile [11]. A classic study investigated whether levels of oxLDL reflect the presence and extent of CAD based on coronary angiography finding in 504 patients. In this study, levels of oxLDL were reported as the oxidized phospholipid content per particle of apolipoprotein B-100 (oxPL:apo B100 ratio). A strong and progressive association between oxPL:apo B-100 ratio with the presence and extent of CAD was demonstrated. OxPL:apo B-100 ratio was an independent predictor of the presence of CAD as confirmed by angiography amongst subjects 60 years of age or younger. Further, by combining this ratio with the cholesterol profile, the odds ratio for predicting underlying CAD in young subjects (<60 years old) was drastically improved. Lp(a) lipoprotein measured in the same cohort was also shown to be correlated with the presence of obstructive CAD, suggesting that the atherogenicity of Lp(a) lipoprotein may be mediated in part by associated pro-inflammatory oxLDL [12]. Similarly, plasma level of oxLDL and antibodies to oxLDL (anti-oxLDL) were measured in a study of 273 individuals comprising patients with CAD and healthy subjects. Levels of both oxLDL and anti-oxLDL antibodies were significantly higher in patients with angina symptoms compared to healthy subjects. Amongst patients with angina symptoms, significantly higher levels of oxLDL and anti-oxLDL antibodies were further observed in patients with unstable angina symptom, compared to those with stable symptoms [13].

Advanced atherosclerotic disease often manifests due to complications from an acute event related to the disruption of atherosclerotic plaques. As the plaque burden within a segment of the artery increases, the lesion may gradually develop features which render it “vulnerable”, with a propensity towards plaque disruption, subsequent luminal thromboembolisation and resulting in downstream organ injury [14]. Further to the association with disease progression and severity of advance atherosclerotic disease states, oxLDL has also attracted much interest for its potential involvement in the development of vulnerable plaques. In a small prospective study, patients with complex coronary plaques conferring vulnerable features asassessed by angiography were shown to have significantly higher levels of oxLDL, supporting an association between oxLDL and plaque instability [15]. Acute myocardial infarction (AMI) is an exemplary clinical event secondary to plaque rupture. In patients presenting with AMI, oxLDL levels were found to be significantly higher than in patients with unstable or stable angina symptoms. In atherectomy specimens obtained from patients with CAD, the surface area containing oxLDL-positive macrophages was significantly higher in patients with unstable angina symptoms compared to those with stable angina [16]. Local blood sampling of the culprit coronary arterial segment under distal occlusion at the time of percutaneous coronary intervention (PCI) performed for acute coronary syndrome (ACS) revealed a significantly higher concentration of plasma oxLDL as compared to the blood collected at the aortic root, upstream of the culprit plaque lesion. This lends strong evidence that oxLDL may be directly released from the vulnerable plaque [17]. The association between oxLDL and the vulnerable plaque phenotype is not limited to the coronary artery. In atherosclerotic plaques retrieved from patients who underwent carotid endartrectomy (CEA), plaque oxLDL was found to be several folds higher than plasma oxLDL. Carotid plaques were classified by immunohistochemistry based on the abundance of macrophages (Mp). Mp-rich plaques contained significantly higher oxLDL than Mp-poor and corresponded with the specific oxLDL antigen (DLH3) positivity of the plaques. Amongst patients with Mp-rich carotid plaques, plasma oxLDL was also significantly higher than in the control subjects [18]. In another study, which examined plaques from 114 symptomatic patients referred for CEA, the humoral immune response represented by the immunoglobulin (Ig) M and IgG against specific oxLDL antigens were determined. Differential plasma Ig profiles were observed between subjects with vulnerable or stable plaque features. Such findings support the concept that immune response against oxLDL epitopes involved in atherogenesis and the level of such circulating antibodies may reflect disease activity in the arterial wall [19]. Taken together, these findings demonstrate the strong association between oxLDL and the underlying diagnosis of atherosclerosis, the severity of the disease process, and even the propensity of clinical complications due to plaque instability. 

## BALANCE BETWEEN ROS AND ANTIOXIDANT DEFENCES IN THE HUMAN VASCULATURE 

### Sources of ROS in the Human Vascular Wall

The state of oxidative stress *in vivo* is governed by the intricate interplay between enzymatic pathways liable for ROS production and the various endogenous or exogenous anti-oxidant mechanisms responsible for ROS elimination. These different tiers of pathways allow the potential for therapeutic modulation of oxidative stress and will be briefly discussed:

In aerobic cells, ROS are formed as intermediates in the redox processes involved in various physiological and pathophysiological processes, such as cellular metabolism, interaction of ionizing radiation with biological molecules, or synthesis by dedicated enzymatic pathways [20]. The chemistry of ROS formation is a complex process and generally begins with the one electron reduction of molecular oxygen (O_2_), resulting in formation of relatively stable molecules such as superoxide anions (O_2_^.-^), hydrogen peroxide (H_2_O_2_), and hydroxyl anions (HO^.^). In addition, superoxide can react with nitric oxide (NO) by a process that is regulated by the rate of diffusion of both radicals, leading to formation of a highly potent oxidant - peroxynitrite (ONOO^-^) [21]. The main enzyme systems responsible for ROS production in the vessel wall include NADPH oxidase (Nox), xanthine oxidase (XO), uncoupled endothelial nitric oxide synthase (eNOS), enzymes of the mitochondrial respiratory chain [22] and others. 

### Endogenous Antioxidants

There are several endogenous enzymes with antioxidant properties, such as superoxide dismutase (SOD), glutathione peroxidase (GPx), catalase, heme oxygenase (HO), paraoxonase (PON) and the thioredoxin (TXN) system. Threre are 3 forms of SODs in humans. SOD3 is the predominant form in the human vascular wall and lowers superoxide concentrations by catalysing the dismutation of superoxide into hydrogen peroxide and oxygen [22]. The enzyme catalase promotes the degradation of hydrogen peroxide to oxygen and water. In ApoE mice, over-expression of catalase and/or SOD have been shown to retard the development of atherosclerosis [22]. GPx exerts antioxidant effects by reducing free hydrogen peroxide to water. In patients with coronary artery disease, the activity of red cell GPx isoform 1 was shown to have prognostic value in addition to that of traditional risk factors [23]. HOs catalyse the first step in heme breakdown to generate biliverdin. This is subsequently converted to bilirubin, which has radical scavenging properties and is able to directly inhibit Nox enzymes [24]. The PON enzymes are associated with high density lipoprotein (HDL) in plasma. They possess peroxidase-like activity and protect against lipoprotein oxidation [25]. The TRX system is a ubiquitous oxidoreductase system present in the endothelial cells and vascular smooth muscle cells (VSMC) and is able to scavenge ROS such as H_2_O_2 _and ONOO^- ^[26]. In addition to antioxidant enzymes, various redox active compounds are found in biological systems and have antioxidant properties. These are compounds of metabolism such as glutathione and bilirubin. 

### Exogenous Antioxidants

Ascorbate (vitamin C) and α-tocopherol (vitamin E) are potent inhibitors of LDL oxidation by ROS scavenging and blockade of the lipid peroxidation chain reaction [27]. Both agents have been observed to increase NO bioavailability. Oral administration of vitamin C or E have both been demonstrated to improve endothelial function in small clinical trials [28], however the beneficial effects on vascular function observed in small clinical trials were not confirmed by large randomized clinical trials such as the GISSI-prevenzione trial [29], the HOPE study [30], or the Heart Protection Study [31]. It has been suggested that oral vitamin E supplementation, especially in the absence of vitamin C co-administration may actually have pro-oxidant effects and that ROS mediated conversion of α-tocopherol to the α-tocopheroxyl radical on the surface of lipoproteins results in formation of lipid peroxidation [27]. Oral supplementation of vitamins has also been reported to negate the efficacy of statin treatment in cholesterol lowering and abolishes statins’ favourable effect on endothelial function and cardiovascular risk [32, 33]. As such, oral supplementation of anti-oxidant as a therapeutic strategy for oxidative stress remains controversial. 

## CIRCULATING MARKERS OF SYSTEMIC OXIDATIVE STRESS IN HUMAN VASCULAR BIOLOGY

The importance of oxidative stress in atherosclerotic cardiovascular disease is highlighted by the observation that increased markers of oxidative stress exist in the presence of risk factors for coronary artery disease, and have predictive value for cardiovascular risk in primary and secondary prevention [34]. Serum lipid hydroperoxides (LOOH) are generated from polyunsaturated fatty acids and represent primarily products of fatty acid peroxidation. Malondialdehyde (MDA) is an end product of lipid peroxidation and is often measured as thiobarbituric acid (TBA) reactive substances (TBARS). Both LOOH and MDA have been shown to be elevated in association with cardiovascular risk factors such as cigarette smoking and diabetes mellitus [35, 36]. The F2-isoprostanes (F_2_-IsoP) are formed by the free radical catalysed peroxidation of phospholipid bound arachidonic acid and released into the circulation. Levels of both urinary and plasma F_2_-IsoP have been shown to correlate with the number of coronary artery disease risk factors [37]. The following section of this review will highlight some of the experimental and clinical studies which utilized these markers of oxidative stress as endpoint measures, and the predictive values of these biomarkers for cardiovascular risks.

### Lipid Hydroperoxides

Polyunsaturated fatty acids (PUFAs) such as phospholipids, glycolipids and cholesterol in biological systems are particularly vulnerable targets of oxidation by ROS, leading to a process known as lipid peroxidation [38]. Excess ROS initiate the process of lipid peroxidation by abstracting H^**+**^ from polyunsaturated fatty acids. The remaining lipid carbon radical (L^**·**^) undergoes molecular rearrangement to form a conjugated diene. This conformation reacts with molecular oxygen to form a reactive lipid peroxyl radical (LOO^**·**^), which may then abstract H^**+**^ from another lipid species to form the non-radical intermediate of lipid hydroperoxides (LOOHs). LOOHs are the primary products of lipid peroxidation, and may undergo iron-mediated electron reduction and oxygenation, to give epoxy-allylicperoxyl radicals (OLOO^**·**^), which trigger the chain reaction of lipid peroxidation [39]. 

Quantification of LOOHs serves as a direct and useful index of the oxidative status of biological system containing rich in PUFA lipids such as cholesteryl esters. Several methods exist for measuring oxidized lipids but most of these assays are neither specific for the hydroxyperoxyl group nor are they stoichiometric [40]. Of the techniques available, high performance liquid chromatography (HPLC) based approaches offer the best sensitivity and specificity. The HPLC approach relies on separation of various LOOHs according to their lipid class. After extraction of LOOH, different post column detection systems can be applied for the determination of LOOH levels. Examples include chemiluminescence (HPLC-CL) [41], electrochemical (HPLC-EC) [42], and coulometric [43] detection systems. The HPLC-CL method has sensitivity down to 1 pmol and good specificity for LOOH, but the assay is time consuming and therefore may not give accurate results due to degradation of LOOH *in vitro* during sample preparation [40]. The addition of antioxidants and quick processing of samples at 4°C can help to minimise this problem, but is often not feasible in a clinical situation [44]. Coulometry is a useful method for measuring LOOH in biological samples such as plasma, especially cholesteryl ester hydroperoxides (CEOOH). Coulometric analysis determines the amount of matter transformed during and electrolysis reaction by measuring the amount of electricity consumed or produced. After extraction from LDL, the level of CEOOH is determined by reverse phase HPLC with the first and second electrode potentials of the coulometric detector set at -10mV and -50mV respectively [43]. To overcome thermal decomposition of certain LOOHs during the detection phase, detection methods such as by UV absorbance has also been developed [45]. 

Apart from HPLC, other platforms available for measurement of LOOH include gas chromatography-mass spectrometry (GC/MS) and the ferrous oxidation-xylenol orange (FOX) method. In GC/MS, lipid extracts are reduced and subsequently transmethylated with sodium methoxide. The hydroxy fatty acid methyl esters are isolated by silicic acid chromatography and derivatized to their trimethylsilyl ethers prior to analysis by gas chromatography-mass spectrometry [46]. In the FOX method, plasma LOOHs is determined by the reaction in which a LOOH reductant, triphenylphosphine, is used to discriminate between the background signal generated by ferric ions and the signal generated by LOOH. The ferric ion indicator dye, xylenol orange, binds to ferric ion to produce a coloured complex with an absorbance maximum which lies between 550 and 600nm. Quantitation of LOOH in the biological samples is feasible because 3 mol of ferric ion is produced per mol of LOOH added [47]. 

Many experimental and clinical studies utilized the measurement of LOOH as a marker of peroxidative damage of membrane lipids and oxidative stress *in vivo*. Suzuki *et al*. investigated the role of ROS in lipid peroxidation and hepatic cellular injury in a murine model of ischemic reperfusion injury. Plasma and liver phosphatidylcholinehydroperoxide (PCOOH) was measured, along with liver transaminases and liver glutathione stores. The authors observed that prolonged hepatic ischemia with reperfusion lead to decreased liver glutathione store, increased serum hepatic transminases and increased PCOOH. These findings suggested that prolonged hepatic ischemia with reperfusion resulted in bursts of ROS production and a resultant increase in membrane lipid peroxidation; that plasma PCOOH level may be a useful biomarker of ROS induced hepatic membrane lipid peroxidation during ischemia/reperfusion injury [48]. 

Given the evidence of cardiac oxidative stress in animal models, Buffon *et al.* investigated whether a brief episode of myocardial ischemia could produce a detectable cardiac oxidative stress in patients undergoing elective PCI. Plasma conjugated dienes (CD), LOOH, and total antioxidant capacity were measured in the aorta and coronary sinus (CS) of patients at set peri-procedural time-points during PCI, in reference to first balloon inflation. Patients with right coronary artery stenosis, which is not drained by the CS, were studied as controls. In the intervention group, CD and LOOH levels were higher in CS than in aorta and increased significantly within the first 15 minutes after balloon induced ischemia. This increase was not observed within the control group nor in the aortic blood samples. Findings from this clinical study added to the experimental evidence that short episodes of myocardial ischemia induce a sustained oxidative stress, which was reflected by LOOH level detectable in the venous effluent of re-perfused myocardium [49].

The cytotoxicity of oxLDL and its specific relation to atherogenesis was first scrutinised by Chisolm *et al*., who undertook purification and identification of the major cytotoxic molecules derived from OxLDL, by using multiple HPLC separations and screening of subsequent fractions for cytotoxicity. Mass spectrometry and nuclear magnetic resonance identified the purified toxin as 7-beta-hydroperoxycholest-5-en-3beta-ol (7beta-OOH-Chol), a molecule which accounted for approximately 90% of the cytotoxicity of oxLDL. The same molecule was also found in fresh human carotid endartrectomy specimens. These results supported the oxidative modification hypothesis that the oxLDLs present in atherosclerotic lesions are able to induce cell and tissue injury [50]. Further to this finding, Brown *et al*. studied 7-hydroperoxycholesterols (7OOHs) formation when LDL was exposed to different in-vitro oxidizing systems, using the normal-phase HPLC method with UV detection. Lipid extracts from carotid endartrectomy were analysed and only trace amounts of 7OOHs were detected using this method, or with the more sensitive HPLC-CL technique. This finding indicated the instability of 7OOH in biological systems and the potential of cells in the artery wall to further metabolise these oxidized sterols [45]. 

PCOOH has also been used as a measure of lipid peroxidation potential in clinical studies looking at the effect of dietary supplementation of anti-oxidants. In a randomized, double-blind, placebo-controlled study of postmenopausal women, beta-carotene was supplemented to the usual daily diet for 3 weeks. *In vitro* production of PCOOH and utilization of plasma antioxidants in the presence of 2-aminopropane hydrochloride (AAPH, a free radical generator) were measured before and after dietary treatment. Plasma beta-carotene level increased significantly in the treatment group compared to the placebo group and was found to significantly reduce AAPH induced PCOOH production as measured by HPLC-CL [51]. Using similar HPLC-CL techniques, Sanaka et al. measured PCOOH levels in a small cohort of uremic patients with diabetic nephropathy undergoing hemodialysis (HD) to assess the therapeutic effect of 500mg α-tocopherol and 600mg probucol daily. Gender and age matched diabetic patients without end stage renal disease, were selected as control subjects. Plasma PCOOH in diabetic patients underoing HD, were significantly higher than control subjects, while the duration of HD in DM patients was strongly correlated to the plasma PCOOH level, The administration of oral antioxidants maintained plasma PCOOH level to baseline levels compared to the non-treatment group [52]. Despite the controversial status of oral antioxidant supplementation on cardiovascular risk, these studies were nevertheless good examples of clinical studies utilizing lipid hydroperoxide as the endpoint measure on the study of plasma antioxidant capacity.

One of the largest clinical studies that addressed the prognostic value of oxidative stress biomarkers in a clinical setting, was the PREVENT (Prospective Randomized Evaluation of the Vascular Effects of Norvasc) Trial. This was a prospective, double blinded clinical study which included 634 patients with documented coronary artery disease (CAD) who were treated with either amlodipine or placebo. Serum samples were collected at baseline and at 12 months intervals during the 3 year study. Serum LOOH levels were measured and correlated with clinical events. LOOH was measured using the FOX method. Baseline LOOH levels correlated with serum levels of soluble ICAM-1 and TBARS. Patients in the highest quartile of baseline LOOH experienced significantly higher risk of non-fatal vascular events (HR = 3.24; P<0.001), major vascular procedures (HR=1.8, P<0.001) and all cause vascular events and procedures (HR=2.23, P<0.001). Amlodipine treatment was associated with reduced cardiovascular events and changes in LOOH levels compared with placebo. The same authors also examined the predictive value of TBARS for cardiovascular events in the same cohort and the findings will be discussed in the coming section [53].

## MDA/TBARS

Lipid peroxides are unstable and decompose to form a series of compounds, including reactive carbonyl compounds. MDA is the by-product of the arachidonate cycle and a principle aldehyde product of lipid peroxidation *in vivo*, that is being widely used as an indicator of oxidative stress in biological systems [54]. The TBA assay is the simplest and most popular method for quantifying lipid peroxidation in biological samples. The assay works on the reaction of TBA with MDA to produce a pink coloured MDA-(TBA_2_) Schiff base adduct. The basic principle involves heating the sample to high temperature (95-100°C) with TBA under acidic conditions to allow the formation of MDA-(TBA_2_) adduct. Molecules of MDA and TBA bind in 1:2 ratio in this thermo energy driven reaction and the amount of pink coloured MDA-(TBA_2_) complex produced can be measured colorimetrically by a spectrophotometer with absorbance at 530-540 nm or fluorometerically using an excitation wavelength of 525nm and emission wavelength of 547nm. For increased sensitivity, the complex can be extracted into an organic solvent such as butanol and measured fluorimetrically. Compared to colorimetric methods, fluorimetric measurements have been shown to be more sensitive and specific than colorimetric measurement [55]. A standard curve can be constructed using MDA prepared by the acid hydrolysis of TEP (1,1,3,3,- tetraethoxypropane) as one molecule of TEP yields one molecule of MDA plus four molecules of ethanol when acidified [56]. TBA may react with other aldehydes in the biological sample and in uncharacterised systems it is usual to refer to the assay of TBA reactive substances (TBAR) as the test may not be not specific to MDA [44]. Apart from directly heating the sample, variations of the TBARS assays techniques have been described. For example, distillation of the samples followed by reaction of the distillate with TBA or by extraction of MDA using aqueous trichloroacetic or perchloric acid and reaction with TBA [44].

Criticisms for the application of TBARS assay to biological samples relate to the issue of specificity. Firstly, TBARS assays measure the MDA generated by decomposition of lipid peroxides in the biological samples rather than the free MDA content of the biological system. Secondly, many different aldehydes are formed in the lipid peroxidation process and aldehydes other than MDA can form chromogens with TBA and form complex with similar absorbance or emission wavelengths [57]. In addition, a variety of TBA reactive “materials”, including sugars, amino acids, and bilirubin is generated and can interfere with the sensitivity of the assay [55]. The iron content of the reagents used for analysis may also interfere with the measurement and the use of EDTA as a chelating agent has been shown to reduce variability of the assay [58]. The sensitivity of the assay can be increased by combining it with HPLC to separate such compounds after the heating stage. However, very delicate sample handling is required hence greatly reduces the throughput of this technique [59]. Finally, MDA does not just reﬂect lipid peroxidation but is also a by-product of cyclooxygenase activity in platelets [60]. Therefore the measurement of MDA level in serum may lead to overestimation of the MDA formation *ex vivo*. 

Several enzyme linked immunosorbance assays (ELISA) have been validated for the measurement of MDA in biological samples and are available as commercialised kits. The ELISA method enables specific determination of MDA using an anti-MDA antibody which is coated to the solid phase. The MDAs in the samples form the antigen-antibody complex and are immobilized. A secondary antibody, traditionally conjugated to Horseradish Peroxidase (HRP), is added to each sample and incubated, followed by addition of a chromogenic reporter substrate and termination of the reaction by addition of an acidic solution. The enzymatic reaction leads to colorimetric change and the intensity of reaction is measured spectrophotometrically. The concentration of MDA in the samples is then determined by comparing against the standard curve of known concentrations. In the case of MDA–LDL ELISA, a combination of an anti-MDA-LDL antibody and an anti-apo B antibody may be used [40]. These ELISA methods have been typically validated against the established methods for measurement of lipid peroxidation products (MDA by HPLC and F2Isop by GCMS) and can have good inter- and intra-assay coefficients of variation [61]. 

The TBARS assay has been applied in clinical studies linking oxidative stress response with cardiovascular risk. Cigarette smokers have higher levels of lipid peroxidation suggesting that the proatherogenic effects of smoking are mediated in part by oxidative damage induced by lipid peroxidation [62]. Lipid peroxidation has also been related to the progression of early carotid atherosclerosis in hypercholesterolemic Finnish men in a subgroup of the Kuopio Atherosclerosis Prevention Study. Amongst other cholesterol oxidation products examined, TBARS was measured fluorometrically and it was shown to be one of the strongest predictors of a 3-year increase in carotid wall thickness in a regression model containing more than 30 variables. The finding gave further support to the association between lipid peroxidation and atherogenesis in humans [63]. In a randomised, double-blind controlled trial, we have shown that short term (3 days) treatment with atorvastatin before coronary artery bypass grafting (CABG) reduces circulating MDA, in parallel to the reduction of vascular superoxide generation in human arteries[64] and veins [65], and suppression of myocardial superoxide generation [66]. These effects were due to a direct reduction of vascular [65] and myocardial [66] NADPH-oxidase activity and the improvement of vascular eNOS coupling [64]. However, the association of circulating MDA with these measures of tissue superoxide generation was rather weak [66], suggesting that extrapolation of circulating MDA levels to biological processes taking place within the vascular wall or myocardium should be made with caution.

In the aforementioned PREVENT trial, the authors also measured plasma MDA levels. To address the potential issue of non-specificity of MDA measurement in serum samples, the authors used a 2 stage process incorporating the classic TBA thermo reaction, followed by separation of TBARS using reverse phase HPLC to separate other TBA reactive materials which have the same absorbance/emission wavelengths as MDA-TBARS. Quantitation of TBARS was performed by both spectrophotometry and fluorometry on an adsobosphere C18 column. At baseline, patients with TBARS levels in the highest quartile had a relative risk (RR) of 3.3 for major vascular events; RR of 4.1 for nonfatal vascular events, and RR of 3.8 for major vascular procedures compared to the lowest quartile. In a multivariable analysis, the prognostic value of TBARS on vascular risk was independent to the other inflammatory biomarkers (IL6, CRP) and classical risk factors of atherosclerosis. This analysis showed an independent effect of TBARS on major vascular events, nonfatal vascular events, major vascular procedures and all vascular events and procedures. Although partially confounded by the use of serum samples, the stringent analytical methods employed by the study improved the credibility of the strong predictive value of baseline TBARS for cardiovascular events in patients with stable CAD, that were independent of traditional risk factors and other established inflammatory biomarkers [67].

### Isoprostanes

F_2_-Isoprostanes (F_2_-IsoPs) are a series of prostaglandin F2α-like compounds produced *in-vivo* by non-enzymatic peroxidation of arachidonic acid esterified in phospholipids and then subsequently hydrolysed to their free acid form by the platelet activating factor (PAF) acetylhydrolase. In contrast to LOOHs and MDAs, F2-IsoPs are chemically stable end-products of lipid peroxidation detectable in all human tissues and biological fluids, including plasma, urine, cerebrospinal and broncho-alveolar lavage fluid [68]. Free F_2_-IsoPs are released from tissue into the circulation and undergo partial metabolism. Both the metabolized F2-IsoPs and their metabolites are excreted into the urine [69]. 8-Iso-prostaglandin (8-iso-PGF_2α_) is an abundant F_2_-IsoP formed *in-vivo* in humans and has both vasoconstrictive and platelet-activating properties [70].

As plasma levels of free F_2_-IsoP in healthy humans are quite low, mass spectrometry techniques may be employed for its measurement. Mass spectrometry coupled to gas chromatography mass spectrometry (GC/MS) rather than liquid chromatography mass spectrometry (LC/MS) is generally preferred because of its greater sensitivity. GC/MS for F_2_-IsoP requires a solid phase extraction procedure followed by gas chromatography. LDL is first isolated by ultracentrifugation and the pellet extracted. The F_2_-IsoP component is then analysed as penta-fluorobenzyl ester and trimethylstilyl ether derivatives by monitoring the [M-181] ions, m/z 569, using GC negative ion chemical ionization/mass spectrometry [40]. The urinary levels of unmetabolizedIsoPs and metabolites of IsoP are much higher, so that both GC/MS and LC/MS have been widely utilized for these measurements. Another reliable method for measuring F_2_-IsoP is stable isotope dilution mass spectrometry. Coupling appropriate chromatography to selectively monitor the specific mass of the F_2_-IsoP ensures that closely related arachidonate metabolites such as prostaglandins do not interfere with quantification [71]. 

F_2_-IsoP can also be measured by ELISA techniques. However, due the limited antigenic structure of F_2_-IsoP, ELISAs are usually based on competitive displacement of a labelled conjugate of F_2_-IsoP from surface bound antibody, rather than by the more selective method of sandwich ELISA. This can lead to the issue of nonspecific antigen-antibody interaction and erroneously high values. Studies comparing the values obtained by ELISA and GC/MS measurements have shown disparate results depending on the antibodies used and pre-analytical factors such as the type of sample matrix used for measurement. Fatty acids that are abundantly present in comparison to F_2_-IsoP may also be released from albumin during protein purification strategies, such as immune-precipitation, and can interfere with the quantification. These factors need to be taken into consideration when interpreting the study findings [71]. 

Plasma levels of free and esterified F_2_-IsoPs appear to be significantly higher in smokers than non-smokers and the levels of both free and esterified F_2_-IsoPs fall significantly after two weeks of abstinence from smoking [72]. The plasma level of F2-IsoP has also been correlated to the lipoprotein status: higher levels of F2-IsoPs were seen in adult subjects with low HDL or high LDL levels [73]. In addition, increased F_2_-IsoP levels were also observed in diabetic patients [74]. In the Genetics of Lipid Lowering Drugs and Diet Network (GOLDN) study, F_2_-IsoP was measured using ELISA to assess the effect of fenofibrate treatment on oxidative stress status. After 3 weeks of treatment (160mg/d), significant reduction in plasma F_2_-IsoP was observed in the patients at the highest tertile of baseline F_2_-IsoP, supporting a role of fenofibrate in reducing oxidative stress in these patients [75]. By using a LC/MS-MS technique, Saenger *et al.* [76] examined the value of urinary F_2_-IsoP in the prediction of myocardial injury in ACS. Urinary F_2_-IsoP was measured in patients presenting to the emergency department with clinical suspicion of ACS, and it was demonstrated that troponin-positive patients had significantly higher urinary F_2_-IsoP compared to troponin-negative patients. Such observations further support the potential applicability of markers of oxidative stress such as F_2_-IsoP, in clinical settings. 

The prognostic values of isoprostanes in cardiovascular morbidity have also been investigated by prospective clinical studies. In a case-control study of 93 CAD patients against age and sex matched control subjects, GC/MS was used to measure urinary excretion of the 8-iso-PGF_2α_and the major urinary metabolites. The level of urinary 8-iso-PGF_2α_ correlated significantly with the number of risk factors for all subjects. Amongst patients with confirmed CAD, higher levels of 8-iso-PGF_2α_ and associated metabolites were observed compared to controls. In addition, 8-iso-PGF_2α_was found to be a novel marker of CAD after adjusting for other traditional risk factors [37]. Further to the association with the CAD disease severity, F_2_-IsoP has also been found to be enriched in direct coronary atherectomy (DCA) specimens obtained from patients with established CAD. Compared to apparently normal internal mammary/radial artery specimens, the DAC specimens contained significantly higher levels of F_2_-IsoP [77]. The behaviour of F_2_-IsoP in the specific setting of ACS has also been addressed. LeLeiko *et al*. measured serum total F_2_-IsoP in 108 patient with ACS by a commercialized ELISA, in addition to the other biomarkers associated with ACS [troponin, high sensitivity C-reactive protein (hs-CRP) and choline] to examine the predictive utility of these biomarkers to short term clinical outcome. F_2_-IsoP and choline levels (but not hs-CRP) predicted 30 day major cardiac adverse events (MACE) with receiver operating curve demonstrating a serum F_2_-IsoP level of 124.5pg/ml to be the cut-off level for 30 day MACE with a negative predictive value of 90%. This observation supports the usefulness of F_2_-IsoP for risk stratification of patients presenting with ACS [78]. 

### NO Breakdown Products

Due to the short life-span of NO in biological samples [79] and the subsequent inability to accurately detect NO radicals, several indirect methods have been used to assess NO production. One such method is quantification of the NO breakdown products nitrite (NO_2_) and nitrate (NO_3_), which are collectively termed NO_x_[80]. The easiest and most commonly used method is the Griess reaction assay, which can be applied to a plethora of biological samples, such as serum, urine, cerebrospinal fluid and cell culture media [81]. First, nitrate contained in the sample must be converted to nitrite; this is usually accomplished in biological samples by cadmium or bacterial nitrate reductase. Next, nitrite reacts with a diazotizing reagent (usually sulfanilamide) to form a transient diazonium salt, which in turn reacts with N-naphthyl-ethylenediamine, giving rise to a stable azo compound with a pink/purple color that can be measured spectrophotometrically. The assay is sensitive, able to measure concentrations of nitrite as low as ~0.5 mM and is available in various commercial kits. NO_x _can also be determined with fluorescent [82] and chemiluminescence [83] assays. However, its use in a clinical setting is questioned, mainly due to the fact that dietary intake of nitrate (mostly derived from proteins) heavily influences the values measured in human biological samples [84].

A summary of the circulating biomarkers for oxidative and nitrosative stress discussed so far can be found in Table **1**.

## EVALUATION OF REDOX STATE IN VASCULAR TISSUES

ROS produced in the human vascular wall, are highly reactive molecules and they usually do not lead to the release of any stable by-products to the circulation. Therefore, the vascular wall (or even individual structures such as the endothelium or the vascular smooth muscle cells) behave as independent compartments in terms of their redox state balance [66]. It is widely accepted that measurement of systemic markers of oxidative stress in peripheral circulation offers limited, if any, information about the true redox state in the vascular wall or inside the human myocardium. Although circulating biomarkers are easily applied in a clinical setting and may have a direct predictive value, the biological significance of their between-patients variability is hard to interpret. Therefore, measurement of redox state at a tissue level is crucial for the understanding of the mechanisms of atherogenesis and also for the identification of novel therapeutic targets. Various methods have been developed over the last few years, enabling direct measurement of ROS formation or quantification of more stable products of the reaction between specific ROS and biological structures. In addition, the evaluation of endogenous antioxidant defence systems at a tissue level provides valuable information regarding the mechanisms of atherogenesis.

### Total Antioxidant Capacity

Total antioxidant capacity (TAC) or total antioxidant status (TAS) is a measure of the cumulative effects of the antioxidants present in a biological fluid or tissue homogenate [85]. It is not a simple sum of all the known and unknown antioxidants contained, but rather an integrated parameter which reflects the complex interactions among these substances and their effect on the redox balance of the sample [86]. 

The first assay for the measurement of TAC, called the total radical trapping antioxidant parameter (TRAP), was described in 1985 [87]. It involved generation of peroxyl radicals in the sample after reaction with 2,2'-azobis dihydrochloride (AAPH) and measurement of the oxygen consumption with an electrode. TAC was determined based on the initial lag phase before increased oxygen consumption, which is due to the presence of antioxidants in the sample. Trolox (a water soluble vitamin E analogue) was used as a standard. Since then, several alternative assays have been developed. The oxygen-radical absorbing capacity (ORAC) uses AAPH as peroxyl radical generator but it is not dependent on lag-time measurements, thus solving several problems present in the TRAP assay. Other assays are based on redox reactions which lead to a colorimetric change, easily assessed spectrophotometrically. Examples are the ferric-reducing ability of plasma (FRAP) assay based on the reduction of ferric to ferrous ion at low pH [88], the ABTS^+^ assay depending on reduction of 3-ethyl-benzothiazoline-6-sulfonic acid [89] and the more recent cupric reducing antioxidant capacity assay (CUPRAC) using the copper(II)-neocuproine reagent in ammonium acetate buffer [90]. Unfortunately, comparisons between studies using different TAC measurement methods are impossible, due to variations in the way antioxidants react in each assay. Several studies comparing technical considerations and sensitivity of different techniques have been published [91].

In reality, total antioxidant capacity is a misnomer; in human plasma, the assays are not able to measure either the activity of endogenous antioxidant enzymes (SOD, catalase etc.) or metal binding proteins [92]. What they do measure is low molecular weight antioxidants, which possess the ability to break oxidation chain reactions, namely the water-soluble urate, conjugated bilirubin, vitamin C and thiols, as well as the lipid-soluble flavonoids, carotenoids and vitamin E. Therefore, while they do not represent the actual total antioxidant capabilities of each individual sample, TAC measurements can serve as an efficient tool for evaluating the plasma effects of antioxidant therapeutic strategies. Indeed, TAC has been used in humans as an indicator of the effect of dietary antioxidants such as black tea [93] and adherence to a Mediterranean diet [94].

Total Antioxidant Capacity assays have been widely used in experimental and clinical settings. In animal studies, low TAC levels have been associated with a variety of conditions, such as exercise-induced oxidative stress [95], diabetes [96] and hypercholesterolemia [97]. In cardiovascular disease, reduced TAC levels in the coronary circulation have been associated with acute ischemic stroke [98], as well as brief episodes of myocardial ischemia in patients undergoing coronary angiography [49]. The use of TAC assessment as a predictive factor for human disease has also been explored. TAC determination through a modified TRAP assay has been reported as a useful *ex vivo* biomarker for atherosclerosis staging in patients with coronary heart disease [99]. 

### Chemiluminescence-Based Assays

The term chemiluminescence describes the phenomenon of light emission as a result of a chemical reaction. This is due to the presence of excited electrons on one of the reaction products, which leads to emission of a photon upon return of the electrons to the stable ground state. This differs fundamentally from fluorescence, where electromagnetic radiation needs to be absorbed by the fluorescent medium, in order for electrons to reach an excited state. In biology a chemiluminescent probe is employed which, upon reaction with a biological free radical, releases a photon that can be detected by a luminometer or scintillation counter. This allows for quantification of free radical generation in the sample [100].

The most commonly used chemiluminescent probe is lucigenin (bis-*N*-methylacridinium nitrate), which is cell-permeable and specific to superoxide radical [101]. The first step in this reaction is the reduction of lucigenin by an O2- radical to the lucigenin radical cation, which in turn reacts with a second O2- radical to form the unstable lucigenindioxetane. This molecule breaks down into two *N-*methylacridone molecules, one of which is electronically excited and emits a photon upon decaying to the stable ground state [102]. Lucigenin is extremely sensitive for the detection of O2-, with the rate constant of the first reaction being ≈10^8^mol/L per second [103], much higher than other assays such as cytochrome C reduction [101].

The validity of the lucigenin-enhanced chemiluminescence assay has been discussed extensively in the past. The main issue is a phenomenon called redox-cycling, which relates to the formation of O2- after reaction of the lucigenin radical cation with molecular oxygen (O_2_), thus leading to an overestimation of the total O2- generated by the sample [104]. This reaction can be observed at all concentrations of lucigenin, though much more strongly so at high concentrations (up to 250 µmol/L) [101]. It also depends on the total levels of O2- present in the sample, with higher pre-existing concentrations of O2- leading to smaller contribution of redox-cycling to the total superoxide generated [101]. Despite these concerns, redox-cycling has been proven to account for an insignificant amount of the total O2- generated in cardiovascular biology experiments, due to the low concentrations of lucigenin used (usually 5 µmol/L and not higher than 20 µmol/L) [105]. This has been confirmed by validating this method against others, such as electron spin resonance (ESR), which appears to provide identical results [106]. ESR also demonstrated that any direct stimulation of O2- production by lucigenin in segments of vascular tissue (when low concentration (5 µmol/L) is being used) has a minor, if any, impact on the results under biologically relevant experimental conditions [107]. It has also been stated that redox-cycling leads to a significant effect in O2- generation only in extreme artificial experimental systems [100]. 

As a cell-permeable compound, lucigenin can be used for simultaneous detection of both intra- and extracellular superoxide production in vascular tissue samples [101]. The assay requires the use of a luminometer or scintillation counter which has been switched to the “out-of-coincidence” mode. First, the background measurement corresponding to the reaction vial containing the buffer and lucigenin is recorded. Then, the sample is added, allowed to equilibrate and the new signal is measured. The counts detected by the luminometer or scintillation counter are usually expressed as relative light units (RLU/second) normalized to wet or dry weight. The assay can be modified to allow for quantification of O2- fraction attributed to each specific enzymatic system present in the vasculature, by using the appropriate substrate or inhibitor. For example, NADPH oxidase activity can be assessed after stimulation with NADPH [108] and/or inhibition with specific inhibitors [109]. The effect of uncoupled eNOS on ROS generation can be determined after inhibition with *NG*-nitro-L-arginine methyl ester (LNAME); elevated levels of uncoupled eNOS will result in a lower average count signal, because inhibition of the uncoupled form of the enzyme will lead to lower O2- formation [110]. Other enzymatic inhibitors that have been used include oxypurinol for xanthine oxidase [111], rotenone for mitochondrial oxidase complex I [112], diphenyleneiodonium (DPI) for all flavin-containing oxidoreductases [113] etc. Apart from intact tissue samples, the assay can also be applied to vascular segments denuded of endothelium [112] as well as whole tissue homogenates and membrane fractions [114], with the use of the aforementioned enzymatic substrates and inhibitors. 

In addition to lucigenin, a number of other chemiluminescence probes have been employed for the detection of other reactive oxygen species in biological samples. One of these is luminol (C_8_H_7_N_3_O_2_) which is non-specific and requires use of free radical scavengers, such as ebselen, superoxide dismutase, catalase or uric acid, in order to attribute the signal obtained to a specific free radical. Luminol, in conjuction with ebselen or uric acid, has been successfully used in human myocardial homogenates [114] and human vascular rings [64, 115]. Another probe is the luminophore coelenterazine, which reacts both with superoxide and peroxynitrite [116]. Therefore, like luminol, it mandates the use of scavengers for calculation of each specific radical. Its use has been reported in intact vascular tissue segments [116]. 

Since their introduction in 1991, chemiluminescence-based assays have been of immense value in cardiovascular research. Results obtained through their use have fundamentally altered the way we perceive the pathophysiological processes regulating most aspects of cardiovascular disease. Lucigenin-enhanced chemiluminescence has documented the key role of oxidative stress in vascular function in atherosclerosis [64, 115]. It has also been employed in studies examining the effects of ROS on cardiomyocytes [117], as well as in pathological processes such as atrial fibrillation [114]. Using the lucigenin/luminol-enhanced chemiluminescence assay, we explored the relationship between myocardial redox state and incidence of post-operative atrial fibrillation in patients undergoing CABG. We demonstrated myocardial basal and NADPH-stimulated O_2_^-^, as well as ONOO-, were independent predictors of this common post-operative complication [118].

In conclusion, chemiluminescence-based assays are inexpensive, easy to perform, reproducible and highly sensitive for the detection of ROS generation in biological samples. The repeatability and specificity of lucigenin-enhanced chemilumine-scence is high, therefore it does not require duplication of the experimental results (especially after treatment with SOD), in contrast to many other techniques [100]. Despite the concerns that were previously described over the contribution of redox-cycling to the generation of O2-, the use of low concentrations of lucigenin generates results which are reproducible and easily replicated by other methods, therefore it is an invaluable tool in cardiovascular research.

### Dihydroethidium Staining & High Performance Liquid Chromatography

Dihydroethidium (DHE) staining is a technique used for the detection of intracellular O2- generation in whole tissue samples. The principle underlying the method was believed to be the reaction of the cell-permeable dihydroethidium with O2-, leading to generation of ethidium which binds to nuclear DNA and exhibits red fluorescence [119]. This was detected by fluorescence microscopy and semi-quantified based on the intensity of the signal. In more recent years, however, it has been shown that DHE reacts specifically with O2- to form a distinct product, 2-hydroxyethidium, whereas the ethidium that is produced from DHE is thought to arise from a variety of other reactions that reflect the total redox status of the cell [120]. Due to the fact that 2-hydroxyethidium has a different molecular weight than DHE and ethidium, HPLC can be employed to accurately detect and quantify the product, which represents the total intracellular generation of O2- [121]. 

DHE staining even without HPLC is a very sensitive technique for the detection of intracellular O2- and when coupled with HPLC, can detect O2- quantities as low as 1 pmol/ mg protein [122]. It is also relatively specific, with minimum cross-reaction with other biological free radicals, such as OH-. It has been shown that the HPLC peak corresponding to 2-hydroxyethidium is largely (although sometimes not completely) abolished by SOD, the use of which gives a more accurate estimation of O2- generation [122]. Incubation of the tissue sample with enzyme inhibitors, such as LNAME and apocynin, can further elucidate the contribution of each enzymatic system to the total intracellular O2- production. However, it has been reported that an excess of cytochrome C or other heme-containing compounds present in the biological sample examined can lead to oxidation of DHE and overestimation of the total O2- generated [123]. This is especially relevant to the study of tissues undergoing heavy apoptosis or mitochondrial damage, which exhibit increased release of the aforementioned compounds. In order to improve the specificity of the assay, each experiment should be duplicated after incubation with a cell-permeable superoxide dismutase, such as polyethylene glycol-conjugated or liposomal SOD, and only the SOD-inhibitable signal be used for analysis [123]. When using fluorescence microscopy alone, care should also be taken not to use wavelenghts lower than 480 nm for excitation and 580-600 nm for emission because it has been reported that such wavelengths can lead to detection of fluorescent by-products of the reaction of DHE with H_2_O_2 _[124]. 

DHE staining has been an extremely useful assay in vascular studies. It is regularly used in conjuction with other methods such as lucigenin-enhanced chemiluminescence to confirm the results, but also because it allows for microtopographic localization of superoxide production. For example, in an animal model of angiotensin II induced hypertension, DHE staining has revealed increased O2- production not only in the intima but also in the medial layer and adventitia [125]. Its use has also revealed a marked increase in O2- production in the shoulder region of human atherosclerotic plaques [126]. 

DHE staining, in conjunction with HPLC, exhibits several attractive advantages. It is a relatively inexpensive, easy to perform technique which requires only minimal training and equipment that is widely available. It allows accurate quantification of intracellular O2-, with excellent sensitivity and specificity. Even without HPLC, confocal microscopy of DHE-stained tissue samples allows for a visual representation of localized O2- generation and semi-quantification. A major disadvantage is the inability to detect other biological ROS, such as H_2_O_2_ and ONOO-. In addition, DHE is susceptible to auto-oxidation after light exposure, therefore the assay must be performed in dim light and the DHE dye stored in dark containers [100]. 

### Electron Spin Resonance

Electron Spin Resonance (ESR), also known as Electron Paramagnetic Resonance (EPR) spectroscopy is an analytical technique that is used to detect and quantify chemical species that possess unpaired electrons, such as free radicals and compounds containing transition metal ions [127]. The basic principles underlying this technique can be compared to those of nuclear magnetic resonance (NMR), with the main difference being the detection of shifts in electron spin rather than nuclear spins. More specifically, an electron placed in an external magnetic field with strength B_o_ will align its inherent magnetic moment either parallel or antiparallel to the field. A parallel alignment corresponds to the lowest energy state of the electron and an antiparallel to the highest. This difference in energy is expressed by the equation *ΔE = g_e_µ_B_B_0, _*where *g_e_* is the electron’s g-factor (which is equal to 2,002 for biological samples) and *µ_B_* is the Bohr magneton constant. An unpaired electron can shift from the lowest to the highest energy state after absorbing electromagnetic radiation of energy equal to *e* = *hf* = *ΔΕ*, where *f *is the frequency of the radiation and *h* is the Planck constant. A typical ESR spectrometer is formed by a resonator cavity surrounded by a pair of electrical magnets and a microwave generator that emits radiation towards the cavity. By maintaining a constant microwave frequency, an increase in B_o_ will proportionately increase the energy gap between the two electron states until the condition *e* = *ΔΕ* is met, at which point electrons from the lowest energy state jump to the highest, thus achieving electron spin resonance. This leads to absorption of electromagnetic energy that can be detected, visualized in a spectrum and quantified. Due to the fact that the unpaired electron magnetic spin is affected by the nuclear spin of the atom it is attached to, a phenomenon known as hyperfine coupling, information about the specific nature of the molecule containing the resonating electrons can be extrapolated from the unique spectrum generated [128]. 

Based on the principles outlined above, application of this technique to biological samples would yield poor results as most of the biological relevant free radicals have an extremely short life span and are impossible to detect. To resolve this issue, the method of “spin trapping” was developed [129]. Spin traps are molecules that bind to free radicals, forming more stable adducts that exhibit unique ESR spectra. Among the first such molecules to be used were nitroso [130] and nitrone compounds [131], which exhibit excellent specificity for various free radicals such as O2- and OH- [132]. However, in whole tissues such spin traps are not effective for measurement of specific oxygen-based radicals. This is due to susceptibility of the nitrone adducts to degradation by endogenous antioxidant mechanisms [100]. 

For this reason, different compounds have been employed that do not act as direct spin traps, but rather serve as "spin probes". Being an oxidation target themselves, they form a more stable ESR active agent with a much longer half-life than biological free radicals. The most effective class of such compounds are the cyclic hydroxylamines, comprising a number of different substances that can be used for ROS detection and quantification in a variety of biological samples [133]. For example, CMH (1-hydroxy-3-methoxycarbonyl-2,2,5,5-tetramethylpyrrolidine) penetrates cellular membranes and can be used to quantify intracellular ROS generation in whole tissue samples [134], whereas 1-hydroxy-2,2,6,6-tetramethylpiperidin-4-yl-trimethylammonium is cell-impermeable and is utilized for quantification of extracellular ROS in tissue samples [135]. CPH (1-hydroxy-3-carboxy-2,2,5-tetramethyl-pyrrolidine hydrochloride) has been used for the detection of ROS *in vivo *[133]. It has also been employed for the quantification of extracellular O2- production in intact cells [134], as well as enzymatic ROS production in whole homogenates and membrane fractions after addition of the appropriate substrate [136]. 

Cyclic hydroxylamines are extremely sensitive for the detection of various ROS, forming stable radicals which do not undergo significant biological degradation and have a half-life of several hours [137]. However, CPH is relatively non-specific, since it reacts with both O2- and ONOO- and displays the same ESR spectrum due to formation of the same nitroxide adduct. This necessitates the use of specific ROS scavengers to allow accurate estimation of the appropriate ESR signal to each biological ROS [138]. Newer hydroxylamines exhibit higher specificity for specific free radicals, such as O2- [135].

Electron spin resonance provides an invaluable tool for cardiovascular research. Apart from confirming the results obtained using other methods, as previously mentioned with regards to chemiluminescence, it is being increasingly used for the detection of ROS production in animal models of vascular disease. For instance, it has been used to explore the role of extracellular vascular SOD, as well as that of NADPH-oxidase derived O2- in the activation of inflammatory pathways in endothelial cells [139]. It has also been employed for the *in vivo* measurement of NO and superoxide production in human endothelial progenitor cells transplanted into a nude mouse carotid injury model [140].

In conclusion, electron spin resonance by using a cyclic hydroxylamine is a sophisticated state-of-the art method for the detection of ROS in biological samples. It exhibits excellent sensitivity and is one of the few analytical methods that can detect and quantify ROS directly. The variety of hydroxylamine spin probes available also allows for accurate measurements in a plethora of different biological samples and intracellular versus extracellular compartments. A major limitation to the application of this technique is the very high cost and space requirements of ESR spectrometers, which constitute their purchase prohibitive for a number of laboratories [100]. Another disadvantage is that the spectrometer needs to be operated by someone who has been specially trained to do so, in order to achieve accurate results. A drawback of the technique itself is the low specificity of several hydroxylamine probes to specific biological ROS, due to the fact that the nitroxide produced is identical for all ROS and generates the same EPR spectrum [137]. Specificity can be improved by specific ROS scavenger, such as SOD or urate, and calculating the production of the individual ROS by subtraction. 

### Assessment of Nitrosative Stress

A method that can be employed to estimate the effect of nitrosative stress on cellular components is the assessment of protein nitrosylation. 3-nitrotyrosine (3-ntyr) is formed after reaction of the amino acid tyrosine with a variety of NO-derived oxidants, serving as a stable marker of protein damage by such radicals [130]. Various methods can be utilized to determine free and protein-bound 3-nitrotyrosine content in biological samples, such as spectrophotometric, immunologic, GC/MS and several HPLC methods [141]. Out of all these assays, GC/MS [142] and HPLC with electrochemical detection [143] are the most sensitive and specific, allowing for accurate quantification of 3-ntyr burden in biological samples. In cardiovascular biology 3-nitrotyrosine has been proposed as a potential oxidative marker for determination of atherosclerosis risk in humans [144]. 3-ntyr levels, both in plasma and specific tissues (vessels and myocardium) have been found to be increased in pathophysiological conditions, such as congestive heart failure [145] and hypercholesterolemia [146].

Despite their obvious practical applications and advantages, the aforementioned techniques represent only indirect methods for determination of NO production and its effect on tissues. In more recent years, there have been efforts to develop methods which allow direct quantification of NO radicals in biological samples. A few examples are NO-specific microelectrodes that detect intracellular generation of NO *in vivo*, in cells and tissue samples (e.g. through the patch-clamp technique [147]). These methods have the disadvantage of trying to detect a highly reactive radical (NO^.^) a shortwhile after its generation, therefore the distance of the probe from the NO^.^source massively changes the results of the reading, introducing high variability and inaccuracy. These methods can be improved by using NO-specific spin-traps for ESR [148], and are particularly interesting options for future research.

### Other Methods

#### Cytochrome C Reduction Assay

Ferricytochrome C is directly reduced by O2- to ferrocytochrome C, which displays increased spectrophotometric absorbance at 550 nm. Due to the non-specific nature of the ferricytochrome C reduction, the experiment must be duplicated in the presence of SOD and only the SOD-inhibitable signal be used for O2- determination [100]. The assay is simple to perform, requires only basic laboratory equipment and has been used extensively with neutrophils, isolated enzymes and cell cultures [101]. However, because of its relatively lower sensitivity when compared to other methods, it has limited, if any, use in cardiovascular studies (where O2- production is low). It is also plagued by concerns over enzymatic and oxidative re-oxidation of ferricytochrome C, leading to underestimation of total O2- generation [101]. 

#### Dichlorofluorescein Diacetate Fluorescent Assay 

This assay is used to assess total intracellular ROS generation, mostly attributed to H_2_O_2_ in cell culture and tissue samples. 2-7-dichlorofuorescein diacetate is a cell permeable compound that is cleaved by intracellular esterases after entering the cell, a reaction which generates 2,7-dichlorofluorescin [149]. In turn, this product is oxidized by various ROS to form the highly fluorescent 2,7-dichlorofluorescein [150]. Results are generated using a fluorescent plate reader. This method suffers from a variety of issues, such as inability to accurately quantify specific ROS, self-propagation of free radical generation through a variety of reactions [151] leading to overestimation of the overall ROS presence in the sample, as well as reliance of the technique on intracellular enzymatic systems that may vary considerably among samples [152]. 

#### Amplex Red Assay

This method has been developed by Molecular Probes and can be employed for quantification of extracellular H_2_O_2_ in tissue samples. Amplex Red (N-acetyl-3,7-dihydroxypgenoxazine) is oxidized by H_2_O_2 _in the presence of horseradish peroxidase to form the fluorescent molecule resorufin, the fluorescence of which can be readily measured [153]. The assay is simple to perform and exhibits high specificity and sensitivity, able to detect quantities of H_2_O_2_ in the picomolar range [154]. It is hindered by the unstable nature of the Amplex Red dye [100] and its inherent inability to detect intracellular ROS generation. 

Table **2** summarizes the advantages and limitations of the different techniques available for vascular reactive oxygen/nitrogen species determination.

#### Gene Arrays Evaluating Oxidative Stress Status in Biological Samples

Traditionally, ROS have been perceived as harmful byproducts of aerobic cellular metabolism, with deleterious effects on living organisms. However, in more recent years this view has shifted towards a much more dynamic physiological and pathophysiological role for ROS in the regulation of signaling cascades and gene expression, with various implications for human disease.

A plethora of cellular enzymatic systems exists that possess ROS generating capacities in physiological conditions. The most striking example is the NADPH oxidase family (Nox1-5 and Duox1-2), the sole purpose of which seems to be the generation of superoxide [155]. In addition, major sources of cellular ROS are the mitochondrial complexes I and III, xanthine oxidase, lipoxygenases, cyclooxygenases, heme oxygenases and others. While certain aspects of this physiological ROS generation are clearly defined, such as production of superoxide by phagocytic NADPH oxidase for host defense, it is only in recent years that attempts have been made to understand the implications of regulated ROS production in intracellular signaling. More specifically, ROS have been found to possess functional post-translational protein modification properties, via their interaction with reactive cysteine residues [156]. For example, H_2_O_2_ can lead to oxidation of the cysteine thiol group (RSH) in an enzyme to form the sulfenic (RSOH) and sulfinic (RSOH2) forms, thus functionally altering the enzyme. This is analogous to the way that phosphorylation of a threonin or tyrosine residue of a protein by a kinase can lead to its activation or inactivation. Additional cysteine modifications by ROS include nitrosylation, glutathionylation and formation of disulfide bonds [155]. Recent estimates suggest that there are well over 500 different proteins possessing cysteine residues with putative modulatory capacity by ROS [157].

In tandem with ROS generating enzymatic systems, cells are equipped with a wide array of enzymatic antioxidant mechanisms, including but not limited to superoxide dismutase, catalase, glutathione peroxidase, peroxiredoxins and thioredoxins. While their role in protecting oxidation targets and reversing oxidative damage has been undoubtedly established, it is being increasingly recognized that these antioxidant systems do not function by indiscriminately scavenging ROS but are implicated in maintaining a much more intricate redox balance homeostasis. For example, interaction of thioredoxin with apoptosis signal–regulating kinase 1 (ASK1) leads to inactivation of the latter and suppression of its pro-apoptotic properties [158]. This interaction is redox-dependent; increased intracellular ROS levels oxidize TRX and unbind it, allowing ASK1 to trigger its signaling cascade. A different example is the selective phosphorylation of peroxiredoxin 1 (Prx1) molecules located near the cellular membrane [159]. This leads to their inactivation and allows NADPH oxidase to locally produce O2- necessary to trigger cysteine modifications. 

Apart from post-translational modifications, increasing evidence suggests that ROS levels influence the expression of a number of genes involved in the regulation of redox state through a negative feedback loop. One of the most extensively studied mediators of this gene expression regulatory system is the transcription factor *Nrf2 *[160]. In physiological conditions *Nrf2 *interacts with its inhibitor *Keap1*, a protein with several reactive cysteine residues [161]. Increased intracellular ROS levels modify *Keap1*, dissociating it from *Nrf2*, with the latter now free to upregulate the expression of a host of different antioxidant enzymes. Similar regulatory mechanisms exist for various transcriptional factors and coactivators, such as the FOXO family [162] and PGC1a [163]. This up-regulation of antioxidant gene expression provides an attractive explanation for the benefits of exercise-induced oxidative stress; for example, exercise-induced upregulation of SOD2 in animals has been shown to exert cardioprotective effects against ischemia/reperfusion injury [164].

It is evident that a holistic approach to studying the cellular redox status should incorporate gene expression profiling for all the candidate genes implicated in redox balance regulation. DNA microarrays provide an invaluable tool for such studies. These microarrays are designed to quantify the expression of genes encoding prooxidant or antioxidant enzymes, as well as genes whose expression is regulated by the main redox-sensitive transcriptional factors. A list of candidate genes for inclusion in these microarrays is presented in Table **3**.

## CONCLUSIONS

The critical role of oxidative stress in atherogenesis is now well defined. Therefore, the development of technically easy and reliable methods to evaluate oxidative stress in clinical settings is particularly important. Measurement of circulating biomarkers of oxidative stress is challenging, since circulation usually behaves as a separate compartment than the individual structures of the vascular wall. Current approaches include the measurement of stable products released by the reaction of ROS and vascular/circulating molecular structures, such as serum lipid hydroperoxides (products of fatty acid peroxidation), plasma malondialdehyde (an end product of lipid peroxidation) or urine (rather than plasma) F2-isoprostanes (product of the peroxidation of phospholipid bound arachidonic acid). Most of these biomarkers have been shown to have a predictive value in cardiovascular disease, although their true biological meaning is unclear. 

On the other hand, quantification of oxidative stress at a tissue level is much more accurate. Lucigenin enhanced chemiluminescence measures accurately superoxide radicals, while the use of DHE (either for in situ staining of vascular segments or in the setting of an HPLC quantification method) are also important techniques for the measurement of superoxide anions. Direct measurement of free radicals, such as electron spin resonance spectroscopy and other methods using electrodes able to detect ROS directly (including NO), are also widely used. Finally, the effect of oxidative stress on gene expression profile through vascular redox sensitive transcriptional pathways is also a good method to evaluate the consequences of oxidative stress in the vascular wall.

In conclusion, measurement of circulating biomarkers of oxidative stress is valuable and many of them appear to have predictive value in cardiovascular disease. However, they do not necessarily reflect intravascular oxidative stress, thus cannot be used either as therapeutic targets or as markers to monitor a pharmacological treatment in the clinical setting. It is important to discover new biomarkers that will be more representative of vascular oxidative stress. These biomarkers should be easy to measure in peripheral circulation, in order to allow their use in clinical settings.

## Figures and Tables

**Fig. (1):**
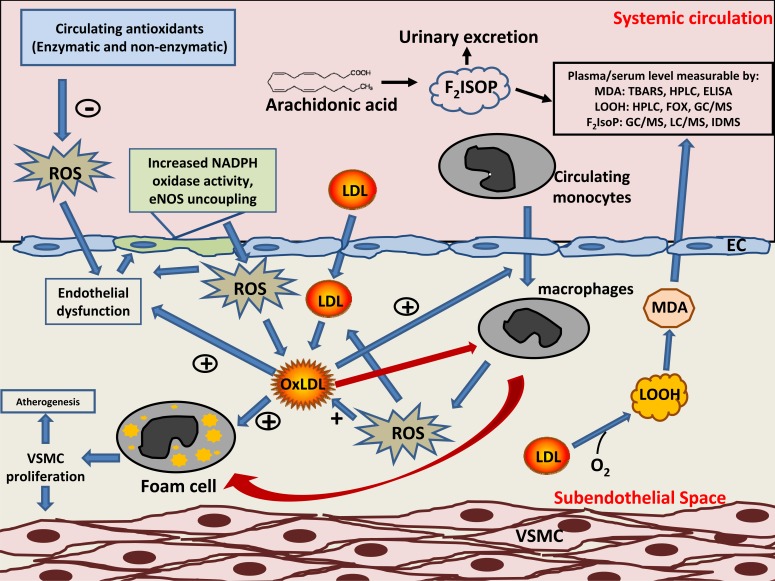
The central role of low density lipoprotein (LDL) and atherogenesis. LDL is protected from oxidative modification in systemic circulation due to the abundant native circulating anti-oxidants. Once trapped in the sub-endothelial space, reactive oxidant species (ROS) reacts with LDL and leads to formation of oxidized LDL (oxLDL). OxLDL is chemoattractant to circulating monocytes and also transcytosed by them via scavenger receptor pathways, leading to the formation of foam cells. Accumulation of foam cells and subsequent vascular smooth muscle cell (VSMC) sequestration/proliferation is the cornerstone of atherogenesis. The chain of events are amplified in a feed- forward fashion as oxLDL contributes towards endothelial dysfunction and further ROS formation. Lipid hydroperoxide (LOOH), malondialdehyde (MDA) and F_2_-Isoprostane (F_2_-IsoP) are products of lipid peroxidation and markers of cardiovascular risk. They can be detected in systemic circulation and measured using various techniques such as high performance liquid chromatography (HPLC), mass spectrometry (MS) or enzyme linked immunosorbance assay (ELISA). (Other abbreviations in the figure: FOX - ferrous oxidation-xylenol orange method; GC-gas chromatography; LC – liquid chromatography; TBARS – thiobarbituric acid reactive substances; IDMS – isotope dilution mass spectrometry; EC-endotheial cell).

**Table 1.** Summary of Analytical Methods of Oxidative/Nitrosative Stress Biomarkers for Blood Samples

Method	Biomarker Measured	Advantage	Limitations
HPLC-CL detection	*LOOH*	Highly sensitive (down to 1pmol) Specific for LOOH	Time consuming, Often not feasible in clinical situations
HPLC with coulometry detection	*LOOH*	Specific for cholesteryl ester -OOH Good for biological samples	Cannot be used for low density lipoprotein (LDL) measurement *in vivo*
GC/MS	*LOOH*	Sensitive	Expensive instrumentation and labour intensive
FOX assay	*LOOH*	Simple protocol Reproducible Sensitive	Issues with specificity and precision due to sample matrix interference (eg. Other pigements, iron chelator proteins) or the media/solvent used for the assay
TBARS assay	*MDA*	Simple, quick protocol Low cost assay	Lack of specificity: other substances in biological samples may also react with TBA to form TBA reactive “materials”
*ELISA*	*MDA*	Easy to performed when available in commercial kit forms	Proprietory reagents and expensive Establishing in-house assays is labour intensive
	*F_2_Isoprostane*	Easy to perform when available in commercial kit forms	Requires extensive purification by chromatography or HPLC prior to assay to avoid interference Sandwich ELISA technique not suitable due to issue with interference.
Griess Reaction, fluorescent and chemiluminescent assays	*NOx*	Sensitive Available in commercial kits Simple to perform	Indirect method of NO assessment Limited clinical use due to influence of results by the patient’s diet

HPLC: high performance liquid chromatography; CL: chemiluminescence; LOOH: lipid hydroperoxide, GC/MS; gas chromatography – mass spectrometry; FOX: ferrous-oxidation xylenol orange; TBARS: thiobarbituric reactive substances; MDA: malodialdehyde; ELISA: enzyme linked immunosorbance assay,NOx: NO breakdown products.

**Table 2.** Evaluating Oxidative Stress Biomarkers in the Human Vascular Wall

Method	Biomarker Measured	Advantages	Limitations
*Chemiluminescence based assays*	O2-, ONOO-	Easy to perform, reproducible, extremely sensitive and specific for O2-, allows for estimation of enzymatic contribution to ROS generation	Concerns over redox-cycling contribution to total superoxide measured, measures other ROS only through use of inhibitors
*DHE Staining*	O2-	Easy to perform, sensitive, allows for topographical localization of O2- generation in vessel layers	Only detects intracellular O2-, duplication after PEG-SOD required for specificity, only allows semi-quantification
*DHE Staining +HPLC*	O2-	Higher specificity than DHE staining alone, allows for accurate quantification of intracellular O2-	Only detects intracellular O2-, does not allow for visualization of O2- generation
*ESR/EPR*	O2-, ONOO-, H_2_O_2_	State-of-the-art method, extremely sensitive and specific, allows direct, *in vivo* detection of ROS	Extremely expensive and space-demanding, requires extensive training, certain probes may not be specific
*3-nitrotyrosine protein content determination*	3-nitrotyrosine protein content	Reflects nitrosative stress damage to proteins	Indirect method for NO assessment, requires GC/MS or HPLC for high specificity and sensitivity
*NO-specific microelectrodes*	NO	Direct detection of NO production in tissue samples, highly sensitive and specific	High intra-assay variability depending on positioning of the probe
*Cytochrome C reduction*	O2-	Simple to perform, once considered the “gold standard” for O2- detection	Limited use in vascular studies due to low sensitivity and specificity
*DichlorofluoresceinDiacetate Fluorescent Assay*	H_2_O_2_	Estimates total intracellular H_2_O_2_ generation	Low specificity, possible overestimation of ROS generated
*Amplex Red Assay*	H_2_O_2_	Simple to perform, high sensitivity and specificity	Trademarked and unstable reagent, only detects extracellular H_2_O_2_

DHE: Dihydroethidium; PEG-SOD: Polyethylene-Glycol conjugated SOD; HPLC: High Performance Liquid Chromatography; ESR/EPR: Electron Spin Resonance/ Electron Paramagnetic Resonance; GC/MS: Gas Chromatography/ Mass Spectrometry.

**Table 3.** Proposed Genes for Inclusion in a Typical Microarray Monitoring “Redox State” in a Human Biological Samples

Biological System	Candidate Gene*	Gene Product Description
***ROS Generating Systems***		
*NADPH oxidase *	NOX2	Catalytic membrane subunit of NADPH oxidase (gp91phox), catalyzing the reaction NADPH + 2H_2_ ↔ NADP^+^ + 2O_2_°- + H^+^; abundant in neutrophils, human endothelial cells, vascular smooth muscle cells, fibroblasts and cardiomyocytes
	NOX1	Nox 1: Homologue of Nox2, present in vascular smooth muscle cells
	NOX4	Nox4: Homologue of Nox2, abundant in vascular smooth muscle cells, endothelial cells, cardiomyocytes and fibroblasts
	NOX5	Nox5: Homologue of Nox2, generates O2- and functions as a H^+^ channel in a Ca^2+^-dependent manner. Present in endothelial cells.
	CYBA	Cytochrome b-245 light chain: The membrane subunit p22phox of NADPH oxidase
	NCF1	Neutrophil Cytosolic Factor 1: The p47phox cytosolic subunit of NADPH oxidase
	NCF2	Neutrophil Cytosolic Factor 2: The p67phox cytosolic subunit of NADPH oxidase
	NCF4	Neutrophil Cytosolic Factor 4: The p40phox cytosolic subunit of NADPH oxidase, interacts with NCF2 to form a complex with NCF1
	RAC1	Rac1: Member of the family of Rho G proteins, interacts with the NCF1-2-4 complex to activate the catalytic subunit of NADPH oxidase
	RAC2	Rac2: Homologue of Rac1
	RHOG	RhoG G-protein: Homologue of Rac1, present in endothelial cells, regulates activation of NADPH oxidase
	DUOX1	Dual Oxidase 1: Homologue of Nox2, located in thyroid and airway epithelial cells
	DUOX2	Dual Oxidase 2: Homologue of Nox2, located in GI tract and salivary glands
	PREX1	Phosphatidylinositol 3,4,5-trisphosphate-dependent Rac exchanger 1: Activates Rac1 by exchanging GDP for GTP
*Mitochondrial Complex I*	>50 different genes	NADH dehydrogenase complex: Oxidizes NADH, transferring electrons to coenzyme Q10. First complex in the electron transport chain. Potent source of superoxide generation in the mitochondria
*Mitochondrial Complex III*	10 genes	Cytochrome bc1 complex: Reduces ferricytochrome C to ferrocytochrome C using dihydroquinone. Third complex in the electron transport chain. Generates superoxide in the mitochondria
*Xanthine Oxidase*	XO	Xanthine Oxidase: Catalyzes the formation of xanthine from hypoxanthine, with simultaneous production of H_2_O2
*Nitric Oxide Synthase*	NOS1	Nitric Oxide Synthase 1 (neuronal - nNOS)
	NOS2	Nitric Oxide Synthase 2 (inducible - iNOS): Located in macrophages and vascular smooth muscle cells. Induced by inflammatory stimuli.
	NOS3	Nitric Oxide Synthase 3(endothelial - eNOS): Located in endothelium, generates NO in its physiological coupled form, generates O2- in its uncoupled form (e.g. after oxidation of its co-factor tetrahydrobiopterin (BH4))
*Lipoxygenase*	7 genes	Human Lipoxygenases: Catalyze the dioxygenation of polyunsaturated fatty acids to form fatty acid hydroperoxides.
*HemeOxygenase*	HO1	Hemeoxygenase 1: Catalyzes degradation of heme to biliverdin, inducible isoform
	HO2	Hemeoxygenase 2: Constitutively active
*Cyclo-oxygenase*	PTGS1 (COX1)	Prostaglandin H2 Synthase 1 (Cyclooxygenase I): converts arachidonic acid to prostaglandin H2 through its dioxygenase and peroxidase ability, constitutively active
	PTGS2 (COX2)	Prostaglandin H2 Synthase-2 (Cyclooxygenase II): normally inactive, its expression is induced by inflammatory stimuli
*Other genes involved in ROS production*	AOX1	Aldehyde oxidase 1: Produces H_2_O2 and possibly O2-
	EPHX2	Epoxide Hydrolase 2: Cytosolic and peroxisomal protein, converts epoxides to dihydrodiols, generating ROS
	MPV17	MpV17 mitochondrial inner membrane protein: Protein involved in the metabolism of ROS
	SFTPD	Surfactant Protein D: Protein which has a role, among others, in ROS generation from phagocytes as host defense
***Redox-sensitive genes***		
*Transcription factors and their regulators*	NFE2L2 (NRF2)	Nuclear factor (erythroid-derived 2)-like 2 : Transcription factor actively involved in the oxidative stress response
	Keap1	Kelch-like ECH-associated protein 1: Inhibits Nrf2 in physiological conditions
	PPARGC1A	Peroxisome proliferator-activated receptor gamma coactivator 1-alpha (PGC1a): Transcriptional coactivator regulating energy metabolism and oxidative stress response. Activated, among others, by ROS and RNS
	FoxO family (1-4)	Forkhead box protein O: Transcription factors regulating the expression of various antioxidant proteins. Triggered by exposure to ROS
*Other ROS responsive genes*	ATOX1	Copper transport protein ATOX1: Copper chaperone with possible antioxidant functions
	CCL5	Chemokine (C-C motif) ligand 5 (RANTES):Chemokine with a role in immune cell recruitment and activation
	HIF1A	Hypoxia-inducible factor 1: Mediates cellular and systemic response to hypoxia
	NLPR3	Cryopirin: Member of the inflammasome, associated with inflammation and apoptosis
	GLRX2	Glutaredoxin 2 (mitochondrial): Participates in various redox reactions
	MSRA	Methionine sulfoxidereductase A: Antioxidant, reduces methionine sulfoxide to methionine
	OXR1	Oxidation Resistance Protein 1
	OXSR1	Oxidative-stress responsive Protein 1
	SCARA (> 10 genes)	Scavenger Receptors: Receptors involved in scavenging oxidized LDL
	SEPP1	Selenoprotein 1: Extracellular glycoprotein with antioxidant functions
	ATM	Ataxia-telangiectasia mutated protein: Kinase activated after DNA damage
***Enzymatic Antioxidant Systems***		
*Superoxide Dismutases (SOD)*	SOD1	Superoxide Dismutase 1: Located in the cytosol and mitochondrial intermembrane space, catalyzes the formation of H_2_O2 from O2-, requires copper and zinc
	SOD2	Superoxide Dismutase 2: Located in the mitochondrial matrix, requires iron and manganese
	SOD3	Superoxide Dismutase 3: Extracellular in homotetrameric form, requires copper and zinc
	CCS	Copper chaperone for superoxide dismutase: Delivers Cu to SOD [Cu-Zn], thus activating it
*Glutathione Peroxidases (GPx)*	GPX1	Glutathione peroxidase 1: Selenoprotein which catalyzes the reaction 2GSH + H_2_O_2_ → GS-SG + 2H_2_O
	GPX2	Glutathione Peroxidase 2: Located in the GI tract
	GPX3	Glutathione Peroxidase 3: Extracellular
	GPX4	PhospholidHydroperoxidase - catalyzes the reaction: 2 GSH + lipid-hydroperoxide → GS-SG + lipid-alcohol + H_2_O
*Peroxiredoxins (TPx)*	PRDX1	Peroxiredoxin 1: Reduces hydrogen peroxide and alkyl hydroperoxides
	PRDX2	Peroxiredoxin 2: Abundant in red blood cells
	PRDX3	Peroxiredoxin 3: Located in the mitochondrial matrix
	PRDX4	Peroxiredoxin 4: Located in the cytoplasm, regulates activation of NF-kappaB
	PRDX5	Peroxiredoxin 5: the gene uses alternate in-frame translation initiation sites to produce 3 distinctly localized isoforms
	PRDX6	Peroxiredoxin 6: Exhibits both peroxidase and lipid hydroperoxidase activities
*Thioredoxin system*	TXN1	Thioredoxin 1: Ubiquitous oxidoreductase enzyme which reduces other proteins via cysteine thiol-disulfide exchange
	TXN2	Thioredoxin 2: Mitochondrial thioredoxin
	TXNRD1	ThioredoxinReductase 1: Cytoplasmic dimeric NADPH-dependent flavoprotein which reduces oxidized thioredoxin
	TXNRD2	ThioredoxinReductase 2:Located in the mitochondria
*Other peroxidases*	CAT	Catalase - catalyzes the reaction 2 H_2_O_2_ → 2 H_2_O + O_2_
	CYGB	Cytoglobin: hexacoordinate hemoglobin with peroxidase functions
	MGST3	Microsomal glutathione S-transferase 3: mediates inflammation and exhibits peroxidase activity towards lipids
*Other antioxidants*	APOE	Apolipoprotein E: Located in chylomicrons and IDLs, its role in the regulation of oxidative stress is increasingly explored
	GSR	Glutathione Reductase: Replenishes cellular GSH by reducing GSSG, requires NADPH
	GSS	Glutathione Synthetase: Forms glutathione from gamma-glutamylcysteine and glycine
	MT3	Metallothionein-3: ubiquitous, low-molecular-weight, cysteine-rich protein with ROS scavenging properties
	SRXN1	Sulfiredoxin-1 : Reduces cysteine sulfinic acid to sulfenic acid in oxidized proteins, protecting them from inactivation

NADPH: Nicotinamide adenine dinucleotide phosphate; GDP: GuanosineDiphosphate; GTP: Guanosine Triphosphate; GSH: Glutathione; GSSG: Glutathione Disulfide; NF-kB: Nuclear Factor kappa B; IDL: Intermediate-density lipoprotein

* The list of these genes may be modified according to the specific characteristics and the expected gene-expression profile of the investigated tissue.
